# Effectiveness of Bio-K+ for the prevention of *Clostridioides difficile* infection: Stepped-wedge cluster-randomized controlled trial

**DOI:** 10.1017/ice.2023.169

**Published:** 2024-04

**Authors:** Jenine Leal, Ye Shen, Peter Faris, Bruce Dalton, Deana Sabuda, Wrechelle Ocampo, Lauren Bresee, Blanda Chow, Jared R. Fletcher, Elizabeth Henderson, Jaime Kaufman, Joseph Kim, Maitreyi Raman, Scott Kraft, Nicole C. Lamont, Oscar Larios, Bayan Missaghi, Jayna Holroyd-Leduc, Thomas Louie, John Conly

**Affiliations:** 1 Infection Prevention and Control, Alberta Health Services, Alberta, Canada; 2 Department of Community Health Sciences, Cumming School of Medicine, University of Calgary, Calgary, Alberta, Canada; 3 Department of Microbiology, Immunology, and Infectious Diseases, Cumming School of Medicine, University of Calgary, Calgary, Alberta, Canada; 4 O’Brien Institute for Public Health, University of Calgary, Calgary, Alberta, Canada; 5 Department of Analytics, Alberta Health Services, Alberta, Canada; 6 Pharmacy Services, Alberta Health Services, Calgary, Alberta, Canada; 7 W21 Research and Innovation Centre, University of Calgary and Alberta Health Services, Calgary, Alberta, Canada; 8 Department of Health and Physical Education, Mount Royal University, Calgary, Alberta, Canada; 9 Department of Medicine, Cumming School of Medicine University of Calgary, Calgary, Alberta, Canada; 10 Calvin, Phoebe, and Joan Snyder Institute for Chronic Diseases, University of Calgary, Calgary, Alberta, Canada; 11 Department of Pathology and Laboratory Medicine, Cumming School of Medicine, University of Calgary and Alberta Health Services, Calgary, Alberta, Canada

## Abstract

**Objective:**

To evaluate the impact of administering probiotics to prevent *Clostridioides difficile* infection (CDI) among patients receiving therapeutic antibiotics.

**Design:**

Stepped-wedge cluster-randomized trial between September 1, 2016, and August 31, 2019.

**Setting:**

This study was conducted in 4 acute-care hospitals across an integrated health region.

**Patients:**

Hospitalized patients, aged ≥55 years.

**Methods:**

Patients were given 2 probiotic capsules daily (Bio-K+, Laval, Quebec, Canada), containing 50 billion colony-forming units of *Lactobacillus acidophilus* CL1285, *L. casei* LBC80R, and *L. rhamnosus* CLR2. We measured hospital-acquired CDI (HA-CDI) and the number of positive *C. difficile* tests per 10,000 patient days as well as adherence to administration of Bio-K+ within 48 and 72 hours of antibiotic administration. Mixed-effects generalized linear models, adjusted for influenza admissions and facility characteristics, were used to evaluate the impact of the intervention on outcomes.

**Results:**

Overall adherence of Bio-K+ administration ranged from 76.9% to 84.6% when stratified by facility and periods. Rates of adherence to administration within 48 and 72 hours of antibiotic treatment were 60.2% –71.4% and 66.7%–75.8%, respectively. In the adjusted analysis, there was no change in HA-CDI (incidence rate ratio [IRR], 0.92; 95% confidence interval [CI], 0.68–1.23) or *C. difficile* positivity rate (IRR, 1.05; 95% CI, 0.89–1.24). Discharged patients may not have received a complete course of Bio-K+. Our hospitals had a low baseline incidence of HA-CDI. Patients who did not receive Bio-K+ may have differential risks of acquiring CDI, introducing selection bias.

**Conclusions:**

Hospitals considering probiotics as a primary prevention strategy should consider the baseline incidence of HA-CDI in their population and timing of probiotics relative to the start of antimicrobial administration.

*Clostridioides difficile* infection (CDI) is recognized as the most important cause of infectious diarrhea occurring in hospitalized patients in developed countries.^1–3^ In the United States, CDI occurred in up to 495,600 patients and resulted in 20,500 deaths in 2017,^4^ with estimated attributable annual costs of $5.4–$6.3 billion.^5,6^ The epidemiology of CDI in Canada is similar.^7^ The principal reservoir for *C. difficile* is the hospitalized patient and the hospital environment, with the risk of acquiring the organism increasing in direct proportion to the length of hospital stay.^8^ The rate of acquisition of *C. difficile* has been reported to be 13% for individuals receiving antibiotics hospitalized from 1 to 2 weeks, increasing to as high as 50% for those hospitalized for >4 weeks.^9^ The risk of CDI further increases with age.^10–12^

Antibiotic exposure occurs in more than half of hospitalized patients,^18^ damaging the microbiome and promoting *C. difficile* colonization, proliferation, and toxin production.^3,13–18^ Measures to reduce the transmission of *C. difficile* include use of private rooms, contact precautions with gloves, gowning and handwashing, environmental hygiene, and antimicrobial stewardship.^15^ Probiotics have been widely accepted as adjunctive measures to bolster the gut microbiome.^19^

Two Cochrane systematic reviews with meta-analyses of randomized controlled trials (RCTs) investigating probiotics for the prevention of CDI have reported that probiotics given during antibiotic therapy can be effective in reducing the risk of developing CDI,^20,21^ but these trials were conducted in settings with high incidence of CDI. Quasi-experimental studies are being used more frequently to assess infection control interventions^22^ for the primary prevention of CDI in the real-world clinical setting,^23^ but most use historic controls as a comparison group. We conducted a pragmatic, stepped-wedge cluster randomized trial (PREVENT CDI-55+) to evaluate the impact of prescribing a probiotic capsule, Bio-K+ (Bio-K International, Laval, Canada) to patients aged ≥55 years who received therapeutic antibiotics in 4 acute-care hospitals in Calgary, Alberta, Canada.

## Methods

### Study population and trial design

**Figure 1.**
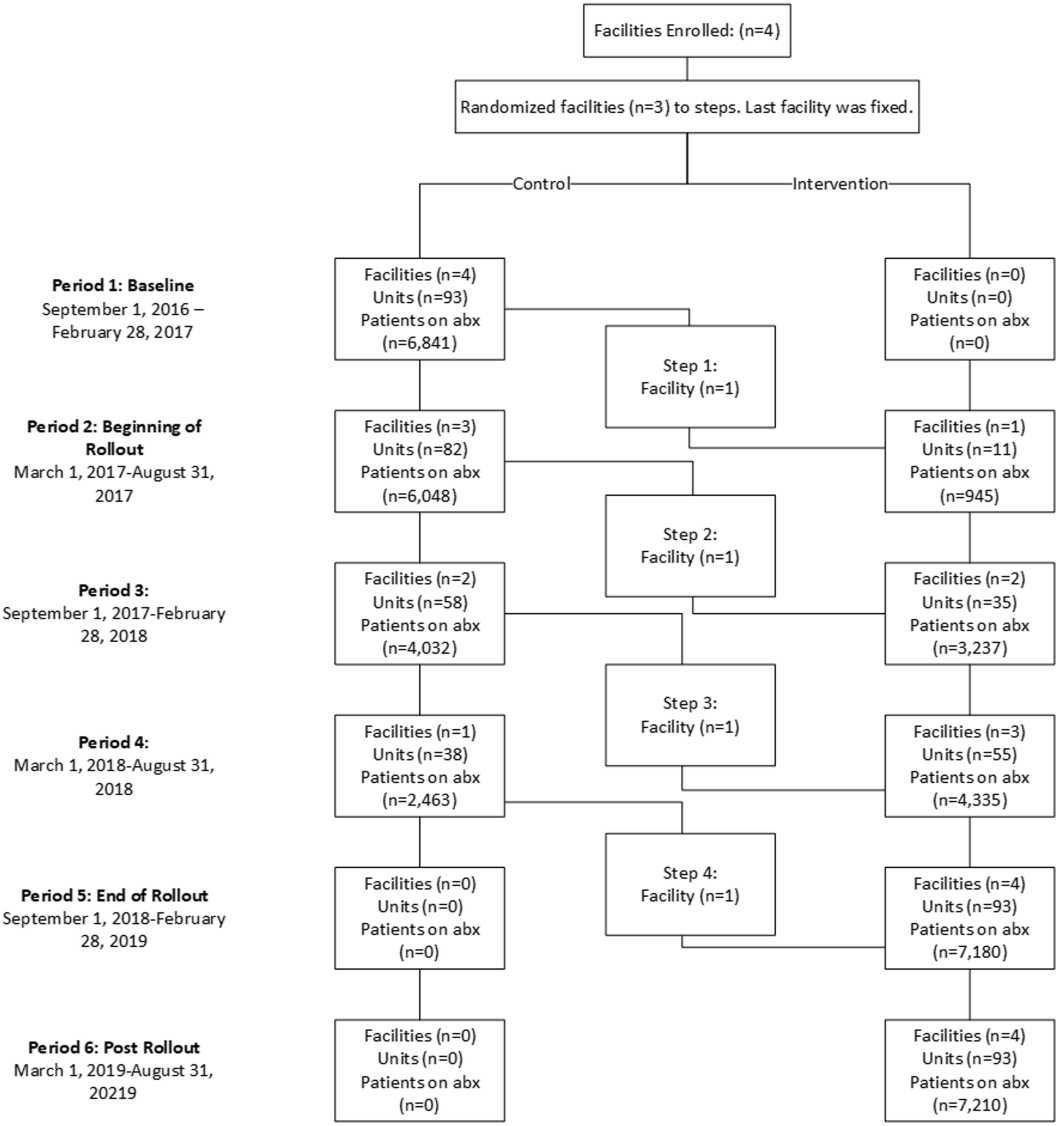
Cluster (facilities) and period flow for the Prevent CDI-55+ study. Patients on antibiotics represents unique patients on antibiotics in each period.

A quasi-experimental, stepped-wedge, cluster randomized trial (SW-CRT) was conducted at the 4 integrated Alberta Health Services acute-care hospitals in Calgary between September 1, 2016, and August 31, 2019. Results were reported according to the CONSORT extension for reporting of SW-CRTs.^24^ The number of acute-care beds at each of these hospitals [South Health Campus (SHC), Rockyview General Hospital (RGH), Peter Lougheed Center (PLC) and Foothills Medical Center (FMC)] ranged from 272 to 1,081. The 36-month study interval was divided into six 6-month periods for each facility, with all facilities starting with a control period. Thereafter, in 6-month intervals, facilities were allocated to start applying the administration of probiotic, with each facility having a minimum 1-year duration of probiotic administration (Appendix 1 online and Fig. 1). Due to concerns that starting the study at FMC as the largest and most complex facility would be logistically challenging, an a priori decision was made to start FMC last, and the remaining facilities were randomly staggered using a random number generator in R version 3.3.1 software (R Foundation for Statistical Computing, Vienna, Austria).

Beginning in May 2016 the alert and accompanying Medical Logic Module for prescribers (ie, attending physicians) was pilot tested over 4 months. Triggered by the order for antibiotics in Allscripts Sunrise Enterprise (Release 15.1), the Bio-K+ line-item provided prescribers direction to order Bio-K+ easily via single-click order entry. To limit ‘alert fatigue,’ the alert would be released once per day, per patient, per prescriber. Pharmacists would review missed opportunities for Bio-K+ ordering daily and adherence to Bio-K+ prescribing was reported monthly to the study team. Prescribers decided on whether to prescribe Bio-K+ based on patient eligibility and clinical judgment. The module only became available to prescribers once the facility began the intervention.

### Participants

Patients aged ≥55 years accounted for 78% of hospital-acquired (HA) CDI cases,^9^ and advancing age is a risk factor for CDI and related complications.^10,25^ Eligible patients were aged ≥55 years admitted to medical, surgical, and intensive care units (ICUs) at each facility, who received systemic therapeutic oral or parenteral antibiotics for >48 hours. Excluded patients were those receiving antibiotic prophylaxis, those admitted to hematology-oncology units receiving active chemotherapy with neutropenia (<1.0 × 10^9^/L) or who were nil per os (NPO) or had ileus. Due to a need to have the simplest medical logic module for antibiotic and probiotic ordering and the possibility that probiotics may mitigate symptoms of CDI and reduce the impact of environmental transmission on HA-CDI, patients admitted to hospital with CDI and who received oral vancomycin or metronidazole monotherapy were allowed to receive probiotic capsules.

### Intervention

Bio-K+ capsules each containing 50 billion colony-forming units (CFU) of *Lactobacillus acidophilus* CL1285, *L. casei* LBC80R and *L. rhamnosus* CLR2 was ordered twice daily, mainly at 10:00 and 22:00, with targeted initiation within 12–24 hours of the first dose of antibiotics, to be continued for 5 days after the final dose of the antibiotics, while in hospital.

### Outcomes

The primary outcome was the incidence of primary HA-CDI cases per 10,000 patient days among hospitalized patients aged ≥55 years as determined independent of the study team by the infection prevention and control program.^25^ Secondary outcomes included (1) severe HA-CDI defined as the proportion of HA-CDI cases that are severe, based on a composite measure of attributable death, colectomy, attributable stay in the ICU stay or colonic perforation within 30 days of HA-CDI diagnosis, (2) *C. difficile* testing rate defined as all *C. difficile* test requests and results (positive, negative, and indeterminate) from inpatients aged ≥55 years per 10,000 patient days, (3) *C. difficile test* positivity rate defined as all positive *C. difficile* specimens collected from inpatients aged ≥55 years per 10,000 patient days, (4) adherence to the intervention, (5) adverse outcomes associated with Bio-K+, and (6) the cost-effectiveness of Bio-K+^26^ for the prevention of HA-CDI over a time horizon of 30 days from the healthcare payer perspective using the decision analysis model by Leal et al.^26^ The *C. difficile* testing rate was applied as a proxy for antibiotic-associated diarrhea (AAD).

### Sample size and power calculations

Assuming the HA-CDI rates were constant at 4 per 1,000 admissions at the 3 major facilities, and 2.6 per 1,000 admissions at SHC, there would be >80% power (α = .05) to detect a 30% relative reduction in rates across all random sequence scenarios, with FMC chosen last. Power simulations were based on 10,000 replications per random sequence scenario.^27^

### Statistical methods

Descriptive analysis was used to compare outcomes in the control and exposed periods. Incidence rate ratios (IRRs) and 95% confidence intervals (CIs) were calculated to compare HA-CDI rates between the control and exposed periods by facility. Test of proportions were used to compare proportions between control and exposed periods by facility. Mixed-effects generalized linear models (GLMs) were used to evaluate the impact of the intervention on HA-CDI rates using a log link and Poisson family function. Mixed-effects negative binomial models were used to evaluate the impact of the intervention on testing rate. During the 6-month study periods, influenza admissions that may impact HA-CDI rates^28,29^ and facility were adjusted for in the models. Facility was entered as a random independent variable, with an unstructured covariance. Likelihood ratio tests were used to assess model goodness of fit. A post hoc analysis including data for 1 year prior to the study period and a 6-month extension of Bio-K+ use was similarly conducted. All statistical analyses were conducted in Stata/SE version 16.1 software (StataCorp, College Station, TX).

### Ethics approval

This study was approved by the Conjoint Health Research Ethics Board at the University of Calgary (no. REB16-1834). Informed consent was not required from individual patients to participate; however, patients received an information package upon Bio-K+ administration containing information on the initiative, use of probiotics, their effectiveness in preventing CDI, and their safety.

## Results

In total, 93 inpatient care units with patients on antibiotics and eligible to receive the intervention were included from the 4 facilities (cluster) enrolled in the study. All units were considered controls during the first 6-month period. Also, 11 units received the intervention in period 2 (March 1, 2017–August 31, 2017), 35 units received the intervention in period 3 (September 1, 2017–February 28, 2018), 55 units received the intervention in period 4 (March1, 2018–August 31, 2018), and 93 units received the intervention in periods 5 and 6 (September 1, 2018–August 31, 2019) (Fig. 1). Each facility received the intervention for at least 1 year. No facility or patient care units dropped out of the study. Prescribers were able to order Bio-K+ for the entire intervention period at their facility.

Total admissions, patient days, and antibiotic courses per period that are not expected to be influenced by the intervention are described in Table 1. There were 269,811 admissions and 1,712,114 patient days at the 4 facilities during the study period. On average, 7,049 patients received antibiotics across the 4 facilities during each period. In total, 49,588 new therapeutic antibiotic orders were made during the study.

**Table 1.** Total Admissions, Patient Days, and Antibiotic Courses Per Period

Period	Sep 1, 2016–Feb 28, 2017	Mar 1, 2017–Aug 31, 2017	Sep 1, 2017–Feb 28, 2018	Mar 1, 2018–Aug 31, 2018	Sep 1, 2018–Feb 28, 2019	Mar 1, 2019–Aug 31, 2019
No. of facilities	4	4	4	4	4	4
Total admissions	42,774	44,095	44,424	45,045	46,453	47,020
Mean admissions	1,782.2	1,837.3	1,851	1,876.9	1,935.5	1,959.2
SD	673.8	693	647.9	709.3	735.7	745.8
Total patient days	291,438.9	285,533	287,264.3	282,600.3	281,223.7	284,053.7
Mean	12,143.3	11,897.2	11,969.3	11,775.0	11,717.6	11,835.6
SD	5,404.8	5,486.7	5,308.9	5,316.3	5,330.5	5,399.4
Patients on therapeutic antibiotics, no.^a^	6,841	6,993	7,269	6,798	7,180	7,210
Therapeutic antibiotic courses, no.^b^	8,317	8,031	8,497	7,863	8,389	8,491
Influenza hospitalizations	425	113	882	100	382	247
Mean	17.7	4.7	36.7	4.2	15.9	10.3
SD	19.1	2.7	34.8	4.7	15.9	9.1

Note. SD, standard deviation.

a Number of unique patients on antibiotics, at any time during the period, across the 4 facilities. Patients could be counted more than once during the entire study period if antibiotic therapy crossed multiple periods.

b A new course of therapeutic antibiotic was counted if the administration date was ≥7 days from the last administration date. Multiple antibiotics prescribed on the same day were counted as a single new course of antibiotic.

### Adherence

**Figure 2.**
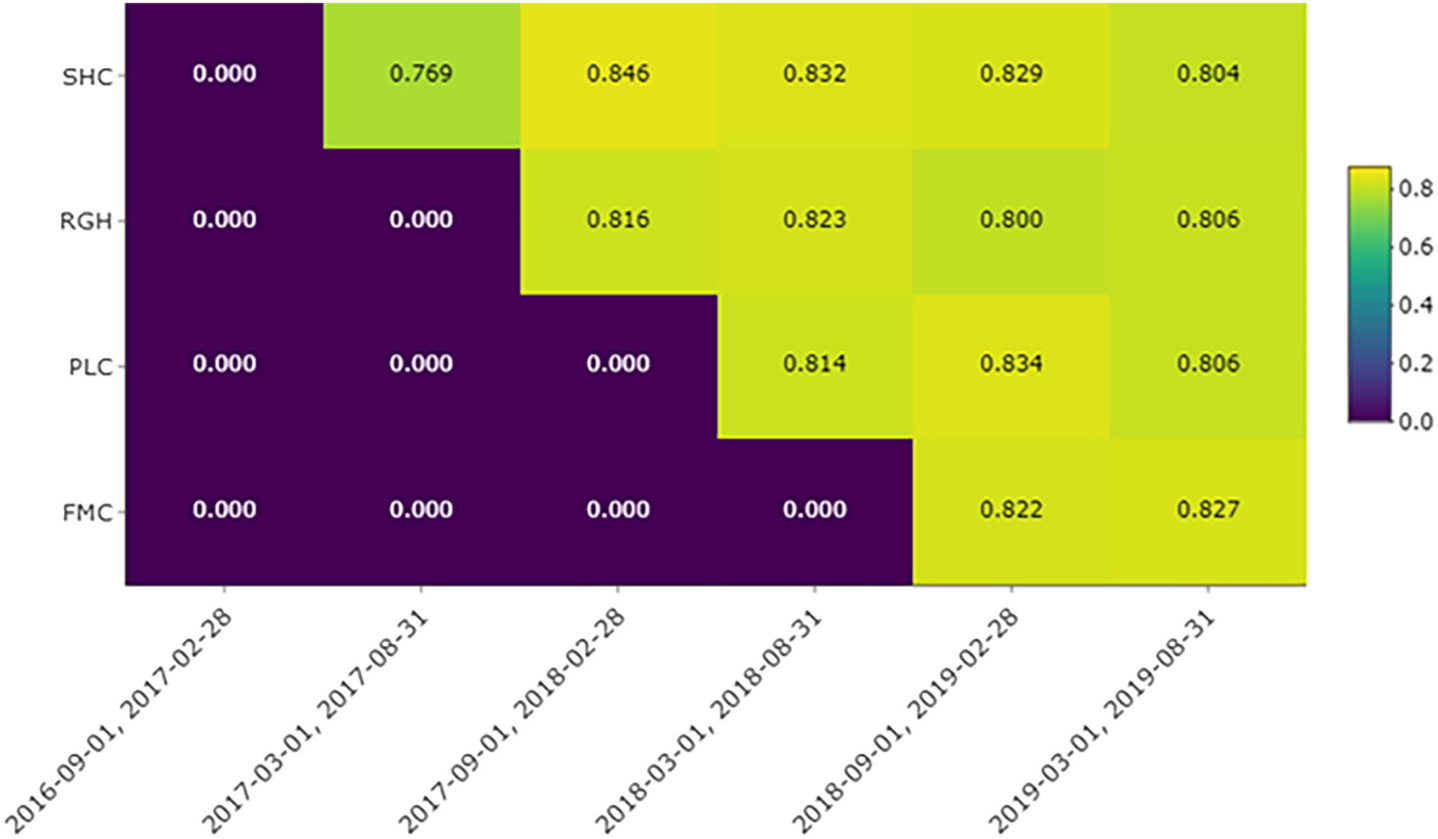
Adherence of the intervention by cluster (facilities) and period. Adherence calculated as the proportion of antibiotic treatments with Bio-K+ administered at any point during antibiotic treatment.

Across the facilities, once the intervention was implemented, 26,727 unique new therapeutic antibiotic treatments were prescribed and 21,824 orders for Bio-K+ capsules, resulting in an adherence rate of 81.6% (Table 1 in Appendix 2). Following the initial period of administering Bio-K+ when adherence was 76.9%, adherence to administering Bio-K+ to patients at any time during their therapeutic antibiotic treatment was maintained between 80% and 84.6% throughout the entire study period (Fig. 2). However, the administration rates of Bio-K+ within 48 and 72 hours of initiating antibiotic treatment were 60.2%–71.4% and 66.7%–75.8%, respectively (Figs. 1 and 2 in Appendix 2). The mean time from antibiotic treatment to Bio-K+ administration was 29.4 hours.

### Unadjusted results of outcomes

**Figure 3.**
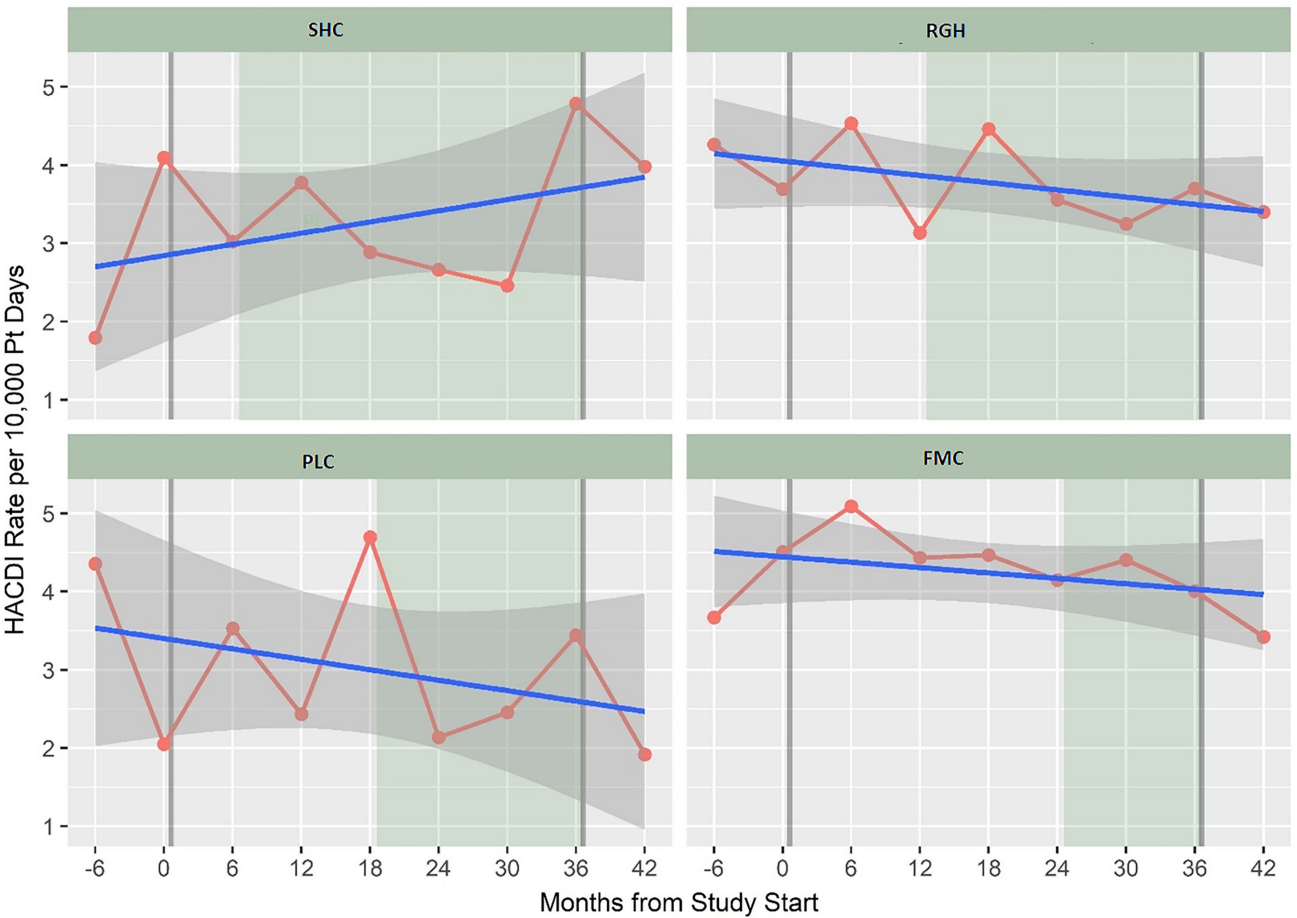
HA-CDI rate per 10,000 patient days by facility and 6-month period between March 1, 2015 (6 months before project start) to February 29, 2020 (6 months after the end of the study period). Linear prediction (blue line) fitted values with 95% confidence intervals (95% CI) (gray) are shown. Gray vertical line indicates the start and end of the study period. The intervention period for each facility is shaded in green.

**Figure 4.**
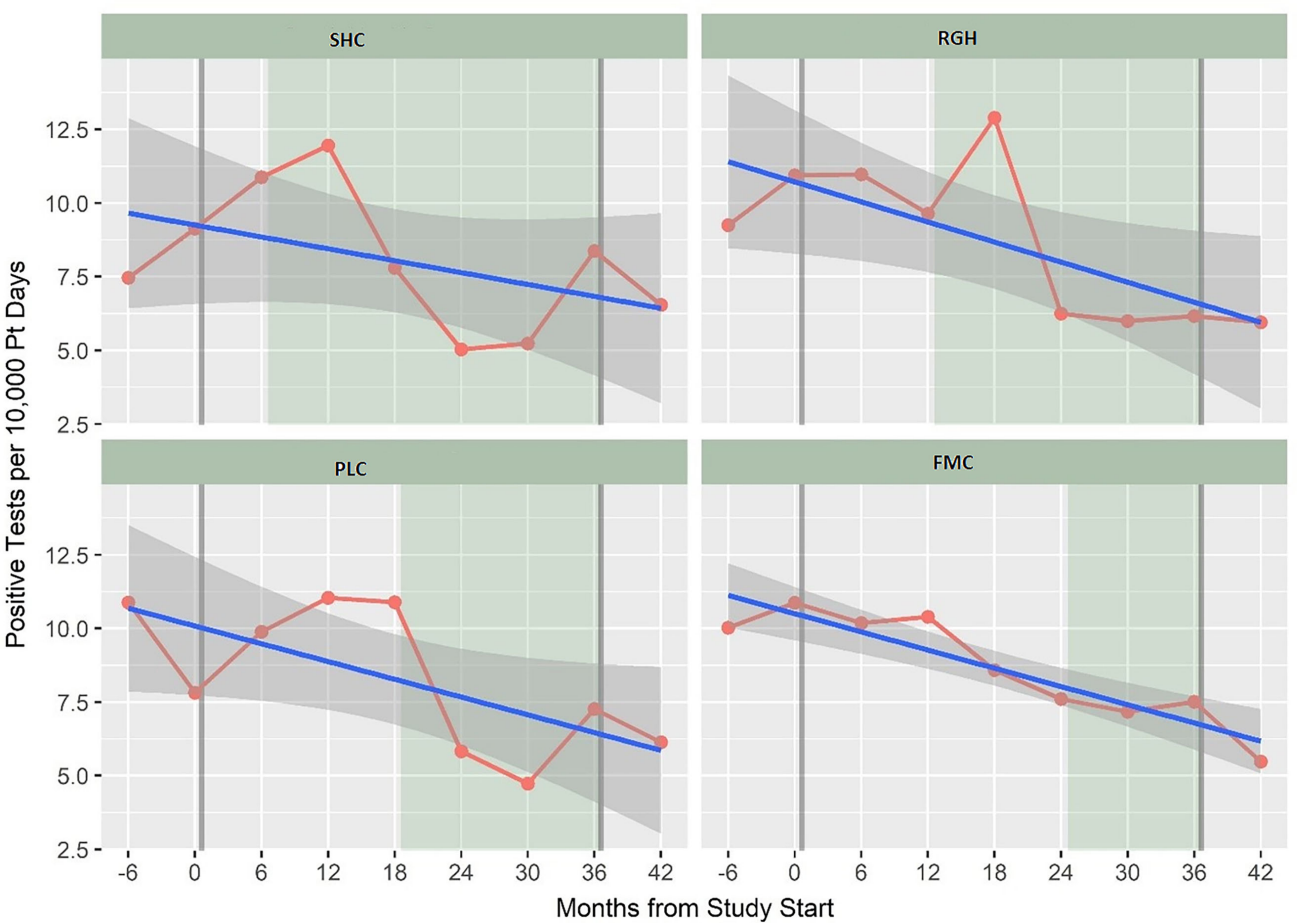
*C. difficile* positivity rate per 10,000 patient days by facility and 6-month period between March 1, 2015 (6 months before project start) to February 29, 2020 (6 months after the end of the study period). Positivity rate numerator is the number of positive *C. difficile* tests among inpatients aged ≥55 years during the study period. Denominator is patient days expressed per 10,000 patient days. Linear prediction (blue line) fitted values with 95% confidence intervals (95% CI) (gray) are shown. Gray vertical line indicates the start and end of the study period. The intervention period for each facility is shaded in green.

Figure 3 shows the unadjusted HA-CDI rate by 6-month period including 6 months prior and after the study. We found a 13.0% overall decrease in HA-CDI rates between the intervention and control periods across all 4 facilities (IRR, 0.87; 95% CI, 0.74–1.01; *P* = .07). We detected a statistically significant 25.0% overall reduction in the positivity rate (all positive tests among inpatients aged ≥55 years) per 10,000 patient days (IRR, 0.75; 95% CI, 0.68–0.83; *P* < .001) across all 4 facilities. The decline in positivity rate was also observed at each facility in Figure 4. Testing rate, as a proxy for AAD, also declined across all 4 facilities (IRR, 0.77; 95% CI, 0.74–0.80; *P* < .001). There was no change in the proportion of positive tests for *C. difficile* across the 4 facilities, despite the decreases in testing. Severe HA-CDI had a relative decrease of 16.0% (*P* = .51). Unadjusted changes in outcomes between the intervention and control periods at each of the 4 facilities are shown in Table 2. There was 1 case of *Lactobacillus* bacteremia during the study, but molecular testing revealed that it was a different strain from the probiotic strains of *Lactobacillus*. Therefore, no adverse bacteremia events were identified during the probiotic intervention.

**Table 2.** Outcomes Between Control and Intervention Periods Across Acute-Care Hospitals

Facility	Variable	Control Period	Intervention Period	Point Estimate (95% CI)	*P* Value
SHC (Cluster 1)	HA-CDI rate^a^	3.02	3.31	1.1 (0.55–2.41)	.82
	Severe HA-CDI %^b^	10.0	7.27	−2.7 (−22.5 to 17.1)	.77
	Testing rate^a,^ ^c^	97.3	72.0	0.74 (0.65–0.84)	<.001
	Positivity rate^a,^ ^d^	10.9	7.6	0.70 (0.48–1.05)	.069
	Positive Tests, %^b,^ ^e^	11.2	10.6	−0.6 (−3.26 to 4.46)	.76
RGH (Cluster 2)	HA-CDI rate	3.84	3.75	0.98 (0.72–1.34)	.87
	Severe HA-CDI, %	4.69	9.02	4.3 (−2.9 to 11.6)	.29
	Testing rate	74.4	58.7	0.79 (0.73–0.85)	<.001
	Positivity rate	10.3	7.9	0.76 (0.63–0.93)	.006
	Positive tests, %	13.9	13.4	−0.47 (−1.98 to 2.92)	.71
PLC (Cluster 3)	HA-CDI rate	3.55	2.68	0.76 (0.49–1.14)	.165
	Severe HA-CDI %	13.79	7.14	−6.6 (−18.4 to 5.2)	.29
	Testing rate	86.9	58.6	0.67 (0.62–0.73)	<.001
	Positivity rate	10.6	5.9	0.56 (0.43–0.72)	<.001
	Positive tests, %	12.2	10.1	−2.06 (−4.65 to 0.53)	.126
FMC (Cluster 4)	HA-CDI rates	4.54	4.21	0.93 (0.72–1.18)	.54
	Severe HA-CDI %	8.96	6.12	−2.8 (−8.9 to 3.3)	.39
	Testing rate	84.1	69.9	0.83 (0.78 to 0.88)	<.001
	Positivity rate	9.2	7.3	0.80 (0.66–0.95)	.011
	Positive tests, %	10.9	10.5	−0.45 (−2.22 to 1.33)	.63

Note. CI, confidence interval; HA-CDI, hospital-acquired *Clostridioides difficile* infection.

a Point estimate for HA-CDI, testing, and positivity rates is incidence rate ratio.

b Point estimate is difference in proportion based on 2-sample test of proportions.

c Testing rate numerator is the number of *C. difficile* tests ordered among inpatients aged ≥55 years during the study period. Rates are expressed per 10,000 patient days.

d Positivity rate numerator is the number of positive *C. difficile* tests among inpatients aged ≥55 years during the study period. Rates expressed per 10,000 patient days.

e Proportion of positive tests is the number of positive *C.difficile* tests among all *C. difficile* tests among inpatients aged ≥55 years during the study period.

### Adjusted analysis

After adjusting for the periods and cluster effects, in the mixed-effects models, HA-CDI decreased by 8.2% during the study period. However, this decrease was not statistically significant (IRR, 0.92; 95% CI, 0.68–1.23). Influenza hospitalization was not statistically significant in the models and was therefore excluded from the adjusted analysis. There was no change in *C. difficile* positivity rate (IRR, 1.05; 95% CI, 0.89–1.24) associated with the intervention after adjusting for periods and cluster effects in the mixed-effects models (Table 3). An underlying period effect with decreasing *C. difficile* positivity rates was observed independent of the intervention. Specifically, decreased rates were observed in period 4 (IRR, 0.61; 95% CI, 0.50–0.75), period 5 (IRR, 0.56; 95% CI, 0.44–0.72), and period 6 (IRR, 0.65; 95% CI, 0.51–0.83) after the 2 largest facilities were enrolled. The post hoc analysis including 1 year prior to the study period and a 6-month extension showed similar results (Table 1 in Appendix 3). The cost-effectiveness analysis suggested that in our setting, the intervention was not cost-effective, with an incremental cost-effectiveness ratio of $129,462 per HA-CDI prevented at a willingness to pay threshold of $80,000 CDN (Appendix 4).

**Table 3.** Unadjusted and Adjusted Results for HA-CDI, Testing Volume, and Overall *C. difficile* Positivity Rates

Hospital	Variable	Control Period	Exposed Period	Unadjusted Incidence Rate Ratio (95% CI)	Adjusted Incidence Rate Ratio (95% CI)
	Patient days	830,578.8	881,535.0	…	…
Overall	HA-CDI rate^a^	4.14	3.6	0.87 (0.74–1.01)	0.92 (0.68–1.23)
	Testing rate^b^	83.2	64.1	0.77 (0.74–0.80)	1.05 (0.96–1.15)^d^
	Positivity rate^c^	9.76	7.3	0.75 (0.68–0.83)	1.05 (0.89–1.24)

Note. CI, confidence interval; HA-CDI, hospital-acquired *Clostridioides difficile* infection.

a HA-CDI rates adjusted for period and cluster (facilities) random effects. Adjusting for influenza admissions did not change the period or cluster effect.

b Testing rate numerator is the number of *C. difficile* tests ordered among inpatients aged ≥55 years during the study period. Denominator is patient days.

c Positivity rate numerator is the number of positive *C. difficile* tests among inpatients aged ≥55 years during the study period. Rates expressed per 10,000 patient days. Adjusted for period and cluster (facilities) random effects.

d Adjusted incidence rate ratio using a mixed-effects negative binomial regression model to account for overdispersion in testing counts. Adjusted for period and cluster (facilities) random effects.

## Discussion

The PREVENT CDI-55+ is one of the largest quasi-randomized studies using the pragmatic stepped-wedge cluster trial design to assess the effectiveness of Bio-K+ for the prevention of HA-CDI. Overall adherence with the intervention was high at >80%, resulting in >21,000 orders of Bio-K+ administered during the study period with no adverse events. A 13% reduction of HA-CDI and 25% reduction in *C. difficile* test positivity rates per 10,000 patient days was observed in the unadjusted analysis. However, the adjusted analysis accounting for period and cluster effects, did not show a statistically significant reduction in our primary outcome of HA-CDI. Although several RCTs^20^ have found probiotics to be effective at preventing CDI, the incidence of CDI in the control groups were higher in those patient populations where significant reductions were observed. A Cochrane systematic review and meta-analysis on probiotics for the primary prevention of CDI found no difference when the baseline risks of developing CDI were 0–2% and 3%–5%.^20^ During the planning of our study rates of HA-CDI were >4.0 per 10,000 days, but as we initiated the study, we found a declining baseline rate of 4.0 per 10,000 patient days (0.25 per 100 admissions), below which it would be harder to detect a reduction in outcome.^15,30^

Maziade et al^31,32^ conducted a 7- and 10-year prospective cohort study in a community hospital in Terrebonne PQ (Montreal), Canada, whereby all adult patients (18+ years) on antibiotics were prescribed Bio-K+. They experienced a 73% reduction in HA-CDI and 76.4% reduction of severe cases.^31^ The reduction in HA-CDI was maintained for 9 years, with rates of HA-CDI stabilizing at low mean levels of 2.3 cases per 10,000 patient days. The decision to introduce the use of Bio-K+ in this hospital occurred during an outbreak when their peak incidence rate was 18.0 cases per 10,000 patient days^31,32^ and when the existing prevention measures were not achieving the desired HA-CDI reductions.

In Canada, HA-CDI rates have declined by 35.8% between 2009 and 2015,^33^ in part due the reduction in the NAP1 strain and the application of infection prevention and control (IPC) measures such as improved diagnostics, environmental cleaning, hand hygiene, public reporting, and antimicrobial stewardship.^33^ During our study period, there were no new IPC interventions; hand hygiene monitoring, antimicrobial stewardship and laboratory testing remained unchanged, limiting confounding of our findings. The downward trend in HA-CDI and *C. difficile* positivity rate observed in both the control and intervention periods with overlap between periods (Appendix 5) may have driven the 13% unadjusted and 8% adjusted reductions in HA-CDI. Other possible reasons for the lack of a significant effect could be not excluding patients who had prehospital antibiotics or antibiotics prior to the intervention. These patients could be colonized by *C. difficile* and at increased risk of HA-CDI. We did not conduct admission screening for *C. difficile* carriage as this is not mandated in Canadian hospitals.

Trick et al^34^ conducted a before-and-after quasi-experimental study using segmented regression to evaluate Bio-K+ for the primary prevention of hospital-onset CDI compared to a 12-month baseline period. The incidence rate was similar during baseline and intervention periods, but they noted a significant decrease in HA-CDI during the final 6 months compared to the first 6 months of the intervention (IRR, 0.6; 95% CI, 0.4–0.9; *P* = .009) despite poor adherence to the protocol.^34^ We observed a similar effect in the secondary outcome of *C. difficile* positivity rate; however, this delayed effect appeared to be independent of the intervention.

Bio-K+ may have reduced the number of viable organisms in the gut, thereby reducing environmental contamination and gradually reducing patient acquisition over time.^34,35^

Our study had many strengths. The study was prospective with Bio-K+ implemented at all adult hospitals across an integrated health region. More than 21,000 Bio-K+ orders were administered to patients, making it one of the largest quasi-randomized studies during a recent period of decreased incidence of CDI, increasing generalizability. The intervention was linked to both electronic ordering and pharmacy, making it easy for prescribers to order Bio-K+ alongside antibiotics. We also had good adherence to the intervention and precise measures to verify bedside adherence. Robust statistical methods were used to account for the study design by including period and cluster effects, and influenza hospitalizations as potential confounders. Without this design and analytical method, we would have observed and reported that the decreased HA-CDI and *C. difficile* positivity rates were associated with the Bio-K+. The primary outcome was measured independently from the study team by the IPC surveillance program using rigorous methods. A cost-effectiveness analysis was also conducted given overall 8% reduction in HA-CDI.

This study had several limitations. We excluded adult patients aged 18–54 years, (31% of the patient days), who may have contributed to the environmental burden and ongoing transmission of HA-CDI. Patients discharged on antibiotics may not have received a complete course of Bio-K+ because it was discontinued upon discharge despite patients being provided instructions on how to acquire Bio-K+ from their local pharmacy. Although there was high adherence with the intervention, the 15%–20% of patients who did not receive Bio-K+ may have had differential risks of acquiring CDI thereby introducing selection bias. Furthermore, adherence was lower than expected within 48 and 72 hours which may have reduced the effects of Bio-K+. Shen et al^36^ found that probiotics were more effective if they were provided closer to the first antibiotic dose, with decrements in efficacy for every day of delay in starting probiotics. Antibiotic-associated diarrhea was not measured, and instead, testing rates were used as a proxy for AAD. Finally, admission screening for *C. difficile* intestinal carriage was not performed in our hospitals.

In conclusion, this stepped-wedge cluster randomized trial with high overall adherence in the use of Bio-K+ did not result in a statistically significant reduction in HA-CDI. This finding may have been due to low baseline HA-CDI incidence and/or delayed initial Bio-K+ administration to patients. Hospitals considering probiotics as a primary prevention strategy for CDI should consider the baseline incidence of CDI in their adult high-risk population and timing of probiotics relative to the start of antimicrobial administration.

## Supplementary material

To view supplementary material for this article, please visit https://doi.org/10.1017/ice.2023.169
